# Identification of a new structural family of SGK1 inhibitors as potential neuroprotective agents

**DOI:** 10.1080/14756366.2022.2153841

**Published:** 2023-01-13

**Authors:** Ines Maestro, Enrique Madruga, Patricia Boya, Ana Martínez

**Affiliations:** a Centro de Investigaciones, Biológicas Margarita Salas-CSIC, Madrid, Spain; b Centro de Investigación Biomédica en Red en Enfermedades Neurodegenerativas (CIBERNED), Instituto de Salud Carlos III, Madrid, Spain

**Keywords:** Protein kinase inhibitors, SGK1, virtual screening, mitophagy, neurodegeneration

## Abstract

SGK1 is a serine/threonine kinase involved in several neurodegenerative-related pathways such as apoptosis, neuroinflammation, ionic channel regulation, and autophagy, among others. Despite its potential role as a pharmacological target against this kind of diseases, there are no reported inhibitors able to cross the BBB so far, being a field yet to be explored. In this context, a structure-based virtual screening against this kinase was performed, pointing out the deazapurine moiety as an interesting and easy-to-derivatize scaffold. Moreover, these inhibitors are able to i) exert neuroprotection in an *in vitro* model of AD and ii) block mitophagy in a PRKN-independent manner, reinforcing the hypothesis of SGK1 inhibitors as neuroprotective chemical tools.

## Introduction

Serum and glucocorticoids-induced kinase 1 (SGK1) is a serine/threonine kinase which belongs to the AGC kinase family and shows similarities with other family members like AKT or PKC. As its name suggests, SGK1 is regulated by levels of serum, and glucocorticoids^1^, being also stimulated by other factors like DNA damage, oxidative stress, excessive glucose concentrations, and so on^2^. This variety of stimuli promotes the translocation of SGK1 between the cytoplasm and nuclei, although it is mainly located at the mitochondrial surface. It is attached to the outer membrane by the N-terminal domain, leaving the C-terminus facing the cytoplasm. Thus, SGK1 stays accessible to be activated by cytoplasmic kinases^3^.

**Figure 1.**
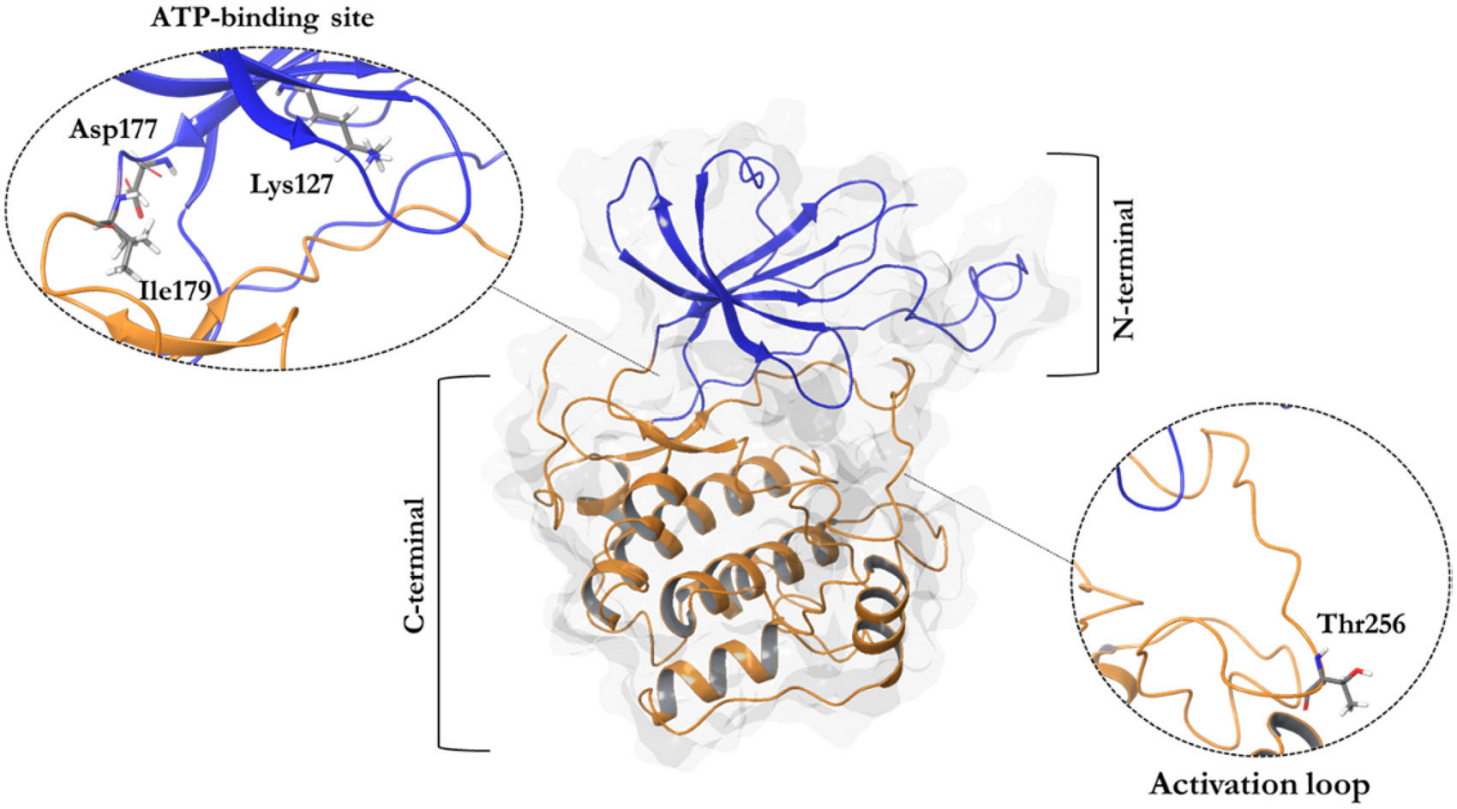
A ribbon diagram of the SGK1 kinase. The C-terminal domain, in orange, shows a α-helical disposition, while the N-terminal domain, in blue, has a β-strand secondary structure. The two domains are linked by the activation loop, where resides the amino acid Thr256, needed for its activation. Residues Asp177 and Ile179, which form hydrogen bonds with SGK1 inhibitors, and the catalytic Lys127, are located in the ATP-binding site.

Structurally, SGK1 shows a classical bilobal kinase fold with the C-terminal α-helical domain (residues 180–431) and the N-terminal β-strand domain (residues 60–179), which are linked by the activation loop (Figure 1)^4^. There are four available crystals of SGK1 characterised with different ATP-competitive ligands. In all these crystals, the inhibitors form hydrogen bonds with the backbone of the hinge region, specifically the carbonyl and amide group of residues Asp177 and Ile179, respectively^5^. To become active, SGK1 needs to be phosphorylated first by the mechanistic target of rapamycin complex 2 (mTORC2) at Ser422, and then by PDK1 at Thr256, being this last residue located in the activation loop of the kinase^5^.

Protein kinases are responsible for modulating the activity of other downstream proteins by means of their phosphorylation of specific residues like serine, threonine or tyrosine. They are found in important cellular processes, like cell proliferation, apoptosis, neuroinflammation and other cell signalling pathways. Pathological imbalance of these protein kinases has been reported in several pathologies, including neurodegenerative diseases. Thus, they have become a perfect target to develop a drug discovery program in order to find new compounds to modulate them^6^^,^^7^. In fact, in December 2021 there were 68 protein kinase inhibitors approved by the FDA, most of them with oncology purposes^8^. However, none of them are directed to neurodegenerative diseases, which are everyday more prevalent in the society due to the increase in life expectancy.

In this case, SGK1 is a kinase involved in the regulation of ionic traffic in different pathological backgrounds, modulating Na^+^, K^+^ and Ca^2+^ concentrations by means of channel regulation. Their role in glucose metabolism has also been reported, as it phosphorylates the glucose transporters GLUT1 and GLUT4, increasing their plasma membrane abundance. In addition, it also participates in the regulation of the immune system by favouring a Th17-pro-inflamatory phenotype^9^. It is worth noting that alterations in this kinase have been found predominantly in cancer. Nevertheless, it has also been described in other pathological conditions, like hypertension, diabetes or neurodegeneration. In fact, its role in neurodegenerative diseases may be particularly relevant as it has been described to be dysregulated in Amyotrophic Lateral Sclerosis^10^, or Parkinson’s Disease^11^. Moreover, SGK1 overexpression in mouse hippocampus triggered neurodegeneration, a decline in cognitive function and an increase of Ser214 phospho-tau protein^12^, which is highly related to tauopathies like Alzheimer’s Disease (AD)^13^^,^^14^.

**Figure 2.**
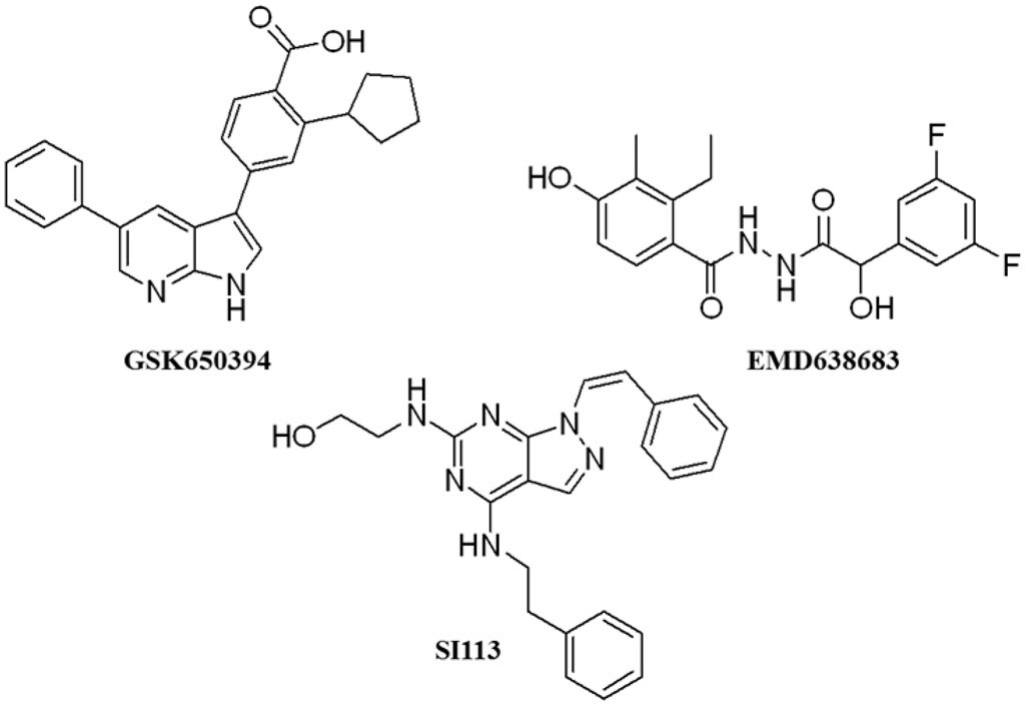
Structure of commercial inhibitors of SGK1.

These data make SGK1 a potential pharmacological target for different pathological situations, especially in neurodegeneration, a field still to be explored. In fact, there are already some SGK1 inhibitors described in the literature. The most used are a pyrrolo-pyridine compound GSK650394 (IC_50_: 62 nM) developed by GlaxoSmithKline^15^, a phenylbenzohydrazide EMD638683 (IC_50_: 3 µM), developed by Merck^16^, and a pyrazolopyrimidine-based compound SI113 (IC_50_: 600 nM)^17^, (Figure 2). However, they have been mainly applied in cancer research and their ability to cross human blood brain barrier (BBB) is unknown. Due to the relevance role of SGK1 in neurodegeneration, its modulation in the CNS must be explored.

In addition, the role of SGK1 in autophagy is being explored^9^. Autophagy is a degradative process, by which cytoplasmic content is sequestered in a double-membrane structure, called autophagosome. The autophagosome will fuse with lysosomes, where the cargo is degraded^18^. As mTORC2 participates in SGK1 activation and inhibits autophagy^19^, SGK1 could be considered as a negative regulator of the pathway^20^. In fact, autophagy was upregulated in muscle in SGK1-deficient mice^21^. Thus, the inhibition of the kinase by the compound GSK650394 induced autophagy^22^^,^^23^. However, a recent work done in HEI-OC1, a mouse auditory cell line, showed an upregulation of SGK1 and autophagy when cells were treated with caffeine. This event was inhibited when cells were co-treated with the inhibitor GSK650394^24^. Thus new studies are needed to unravel the autophagy modulation by SGK1.

To try to understand the role of SGK1 in autophagy, validated phenotypic assays to study the selective degradation of mitochondria by autophagy, named as mitophagy, are useful^25^. Mitophagy degrades specifically mitochondria using regulators of the autophagy machinery. There are several types of mitophagy, the most known are PRKN-dependent and -independent pathways. PRKN is an E3 ubiquitin ligase, which marks the surface of the mitochondria with residues of ubiquitin that are recognised by autophagy machinery to be degraded. The second pathway is independent of the ubiquitination events and relies on the ability of receptors to recruit mitochondria to growing autophagosomes. These pathways are precisely regulated but alterations have been described in different pathological conditions, including some neurodegenerative diseases^26^.

With all these in mind, the goal of this work is to find new SGK1 inhibitors permeable to BBB in order to explore the role of this kinase in mitophagy modulation with the future perspective of being used to treat neurodegenerative diseases.

## Material and methods

### Blood brain barrier (BBB) penetration prediction in silico

BBB penetration was estimated for each compound according to SwissADME parameters (http://www.swissadme.ch/)^27^. A precise description of the performed calculations can be consulted elsewhere^28^. Briefly, the BOILED-egg model combines lipophilicity (wLogP) and polarity (PSA) of the desired compounds to classify them according to their predicted gastrointestinal and brain penetration with high accuracy.

### Virtual screening

#### Protein preparation

SGK1 crystallographic structure 3HDM^5^ was obtained from Protein Data Bank database (PDB). The structure was prepared with Protein Preparation Wizard module (Schrödinger)^29^. The system was pre-processed by default. The ligand, metals and molecules of water were removed, and hydrogens were included. Residues were ionised at a pH 7.2 and minimised with OPLS2005 force field.

#### Ligand preparation

MBC library was prepared with Ligprep (Schrödinger)^30^. Hydrogens were added, protonation states and tautomers were simulated at pH 7.2 ± 2 with Epik, salts were eliminated, and ligand geometry was optimised through a minimisation with the OPLS2005 force field.

#### Docking methodology

For grid generation and docking, Glide (Schrödinger) was used^31^. The docking site was defined based on the position of the ligand crystallised in the catalytic site.The MBC library was screened in XP precision mode. Compounds with hydrogen bonds with Asp177 and Ile 179, like the control reference, were considered. Results were order based on the docking score (XP Gscore).

### Compound preparation

All the compounds, coming from our in-house MBC library,^32^ were prepared with a stock concentration of 25 or 10 mM in DMSO. The final % of DMSO in cell culture was not higher than 0.1%. Bafilomycin A1 (Baf1) (Enzo Life Sciences – BML-1100–0100) and okadaic acid (OA) (Sigma – O9381) were also prepared in DMSO. Deferiprone (DFP) (Sigma − 379409) was dissolved in water. Carbonyl cyanide-p-trifluoromethoxyphenylhydrazone (FCCP) (Sigma, C2920) was dissolved in ethanol.

### SGK1 inhibition assay

Assays of kinase inhibition were done in the MRC Unit of Dundee University, under the following method: SGK1 (5–20 mU diluted in 20 mM MOPS pH 7.5, 1 mM EDTA, 0.01% Brij35, 5% glycerol, 0.1% b-mercaptoethanol, 1 mg/mL BSA) was assayed against a modified Crosstide peptide GRPRTSSFAEGKK in a final volume of 25.5 µL containing 8 mM MOPS pH 7.0, 0.2 mM EDTA, 30 µM substrate peptide, 10 mM magnesium acetate and 0.02 mM [33P-g-ATP] (50–1000 cpm/pmol) and incubated for 30 min at room temperature. Assays were stopped by addition of 5 µL of 0.5 M (3%) orthophosphoric acid and then harvested onto P81 Unifilter plates with a wash buffer of 50 mM orthophosphoric acid.

### Cell lines

U2OS-iMLS (± PRKN) FlpIn TRex cells. Human osteosarcoma U2OS cell line with stable inducible expression of the internal mitochondrial localisation signal (iMLS) (NIPSNAP1_1-53_)-EGFP-mCherry^33^. They were generated with the FlpIn system using the pcDNA5-MLS-EGFP-mCherry plasmid. As those cells do not express PRKN, U2OS-iMLS were transduced with a lentiviral particle expressing PRKN. Both cell lines were culture with DMEM with glutamine supplemented with 10% FBS and 1% Pen-Strep at 37 °C and 5% CO_2_. Cells were selected with 100 µg/mL hygromycin and 5 µg/mL blasticidin. Additional 2 µg/mL puromycin were used for U2OS-iMLS-PRKN cells. To induce the expression of the mitophagy reporter, 500 µg/mL doxycycline was added for the last 24 h. These cells were obtained from Prof. Anne Simonsen Lab (Faculty of Medicine, Institute of Basic Medical Sciences and Centre for Cancer Cell Reprogramming, Institute of Clinical Medicine, University of Oslo, Norway).

SH-SY5Y MitoQC. Human neuroblastoma cell line stably expressing the mitophagy reporter mCherry-GFP-FIS1_101-152_^34^. This cell line was maintained in DMEM:F12 (1:1) supplemented with 15% de FBS, 1% glutamine 2 mM and 1% de pen-strep (0.5 mg/ml) at 37 °C and 5% CO_2_. MitoQC reporter was obtained as follow: mCherry, GFP and FIS1_101-152_ residues cDNA were cloned into a pBABE.hygro vector.^34^ The construct was co-transfected into 293FT cells with GAG/POL and VSV-G expression plasmids (Clontech, Saint-Germain-en-Laye, France) for retrovirus production using Lipofectamine 2000 (Life Technologies) in accordance with manufacturer’s instructions. Virus was harvested 48 h after transfection and applied to cells in the presence of 10 µg/mL polybrene. Cells were selected with 500 µg/mL hygromycin, and stable pool used for experiments. In order to maintain in culture only the cells with reporter, 500 µg/mL hygromycin are used for SH-SY5Y MitoQC. This cell line was obtained from Dr. Ian Ganley Lab (School of Life Sciences, University of Dundee, Scotland).

### Mitophagy assay

U2OS-iMLS ± PRKN and SH-SY5Y MitoQC cells were seeded at a final concentration of 50,000 and 100,000 cells/mL respectively, in crystals in a 24-well plate. Next day, the cells were treated as indicated. In the case of U2OS-iMLS ± PRKN, 500 µg/mL doxycycline was added to medium to induce the expression of the reporter. After 24 h, the cells were fixed with 3.7% N-(2-hydroxyethyl)piperazine-N′-ethanesulfonic acid (PFA) 200 mM Hepes pH 7 and incubated with DAPI (Sigma-Aldrich─2D9542) to stain the nuclei. Then, the crystals were mounted over slides with ProLong Diamond Antifade Mountant (Thermo, P36961). The slides were kept for 24 h at RT and then at 4 °C until images were acquired.

Images of the U2OS-iMLS ± PRKN cells were obtained with an AF6000 LX widefield multidimensional microscopy system and images of the SH-SY5Y MitoQC cells were obtained with the CLSMLEICA TCS SP8 STED 3X placed in the confocal laser and multidimensional microscopy *in vivo* facility at CIB Margarita Salas (Madrid, Spain). Five to six images were manually taken at 40x and 63x, respectively, to have around 100 cells per coverslip.

Image analysis was done with CellProfiler^35^. In order to identify red-only structures per cell, segmentation of nuclei, cells and mitochondrial network were performed. Mitochondrial structures were further filtered as “yellow” or “red-only” based on the ratio between their EGFP/GFP and mCherry integrated intensities. The final number of red-only structures per cell was used as a mitophagy rate readout. Later, this pipeline was modified in order to analyse mitophagy in cells seeded in 24-well plates. The final number of yellow structures per cell was used to measure mitochondrial mass.

### Citrate synthase assay

A total of 1,000,000 cells/mL were seeded in a 6-well plate. After 24 h, the cells were treated as indicated. Then, the cells were washed with cold PBS 1×. The cells were resuspended in NP-40 lysis buffer in water (150 mM NaCl, 1% NP-40, and 50 mM Tris pH 8), rotated in a cold room for 30 min, and centrifuged at 13,000 g for 20 min in cold. Then, the supernatant was collected and stored at −20 °C until used.

Citrate synthase activity was determined by incubating 5 µL of proteins with 995 µL of 100 mM Tris pH 8, 0.1% Triton, 0.1 mM acetyl-CoA (Sigma, A2181), and 0.2 mM 5,5′ dithiobis(2-nitrobenzoic acid) (Sigma D8130-1G). 198 µL of the mix was pipetted in triplicates on a 96-well plate, and 2 µL of oxaloacetate (Sigma O4126-1G) was added to the sample wells. Absorbance was measured at 412 nm every 30 s for up to 60 min at 30 °C. The results were normalised to protein concentration.

### Viability assay. Crystal violet

Cell death was evaluated with crystal violet, a dye that binds to DNA and protein of remaining adherent cells. 300,000 cells/mL were seeded in a 96-well plate. Next day cells were treated as indicated and 1 h later OA was added to induce cell death. To stop the treatment, cells were washed with PBS 1X and fixed with PFA 3.7% Hepes 200 mM for 30 min. Then, 0.1% crystal violet (Sigma, C0775) in distilled water was added for 30 min at RT in a shaker. Plates were carefully washed with distilled water to remove crystal violet in excess and faced down to dry overnight. Next day, cells were resuspended in 10% acetic acid (Merck) in distilled water. Absorbance at 590 nm was measured and quantified in a plate reader.

### Statistics

Statistics was done with the software GraphPad Prism 7. Analysis was done with the unpaired two tailed t-test to compare two groups or one-way ANOVA followed by Dunnett’s multiple comparison test if there are more than two groups. A *p* values lower than 0.05 was considered statistically significant.

## Results and discussion

### BBB penetration prediction of SGK1 inhibitors

**Figure 3.**
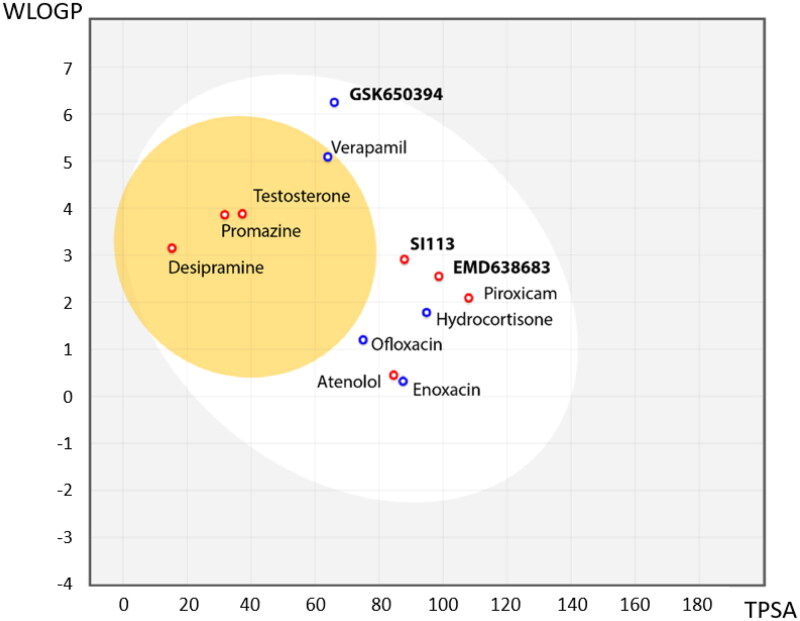
BBB penetration of SGK1 inhibitors according to the BOILED-egg model. The white region corresponds to the chemical space with highest probability of exert good gastrointestinal absorption, while the yellow region corresponds to the chemical space with highest probability to reach the CNS. WLOGP: Wildman and Crippen log P (n-octanol/water partition coefficient); TPSA: topological polar surface area.

As we have described before, SGK1 inhibitors have been mainly tested in non-neurological context such as cancer or cardiac pathologies. This is not a trivial issue, since it is well known that neuroprotective drugs must present sufficient brain penetration in order to exert their effects. As far as we know, there is no data reported about BBB penetration of the already known SGK1 inhibitors, which could explain the lack of experiments in this field. In this work, we used the SwissADME tool of the Swiss Institute of Bioinformatics to predict *in silico* the probability of the described SGK1 inhibitors to cross the BBB. The test was validated with different drugs known to be BBB penetrant (desipramine, peroxicam, testosterone, verapamil) or non-penetrant (atenolol, ofloxacine, hydrocortisone, piroxicam and enoxacin)^36^ (Figure 3(A)). Then, the inhibitors of the kinase were included in the study, showing the inability of the compounds GSK650394, EMD638683 and SI113 to reach the CNS (Figure 3(B)). Thus, a program to find new SGK1 inhibitors that cross the BBB was initiated.

### Compound selection

For compound selection, the Medicinal and Biological Chemistry (MBC) library^32^ was used. It counts on more than two thousand compounds with structural diversity, mainly with heterocyclic scaffolds and enriched in BBB permeable compounds. It has been widely used in different medicinal chemistry programs over the last 20 years and their drug-like properties have been well characterised. Therefore, it was a perfect starting point for the identification of molecules to target SGK1.

First, we followed a target-based drug discovery approach, where a virtual screening against this kinase was performed with compounds from the MBC library. With this strategy, we expected to get rid of the compounds that are predicted to not fit well in the active site according to the scoring function. At the moment of the screening, there were only three available crystals in PDB (3HDM, 3HDN and 2R5T). We selected the 3 D coordinates corresponding to the crystal structure of SGK1, named 3HDM, based on the nature of the ligand, a small heterocyclic molecule, crystallised in the catalytic site and the lower resolution of the crystal.

**Figure 4.**
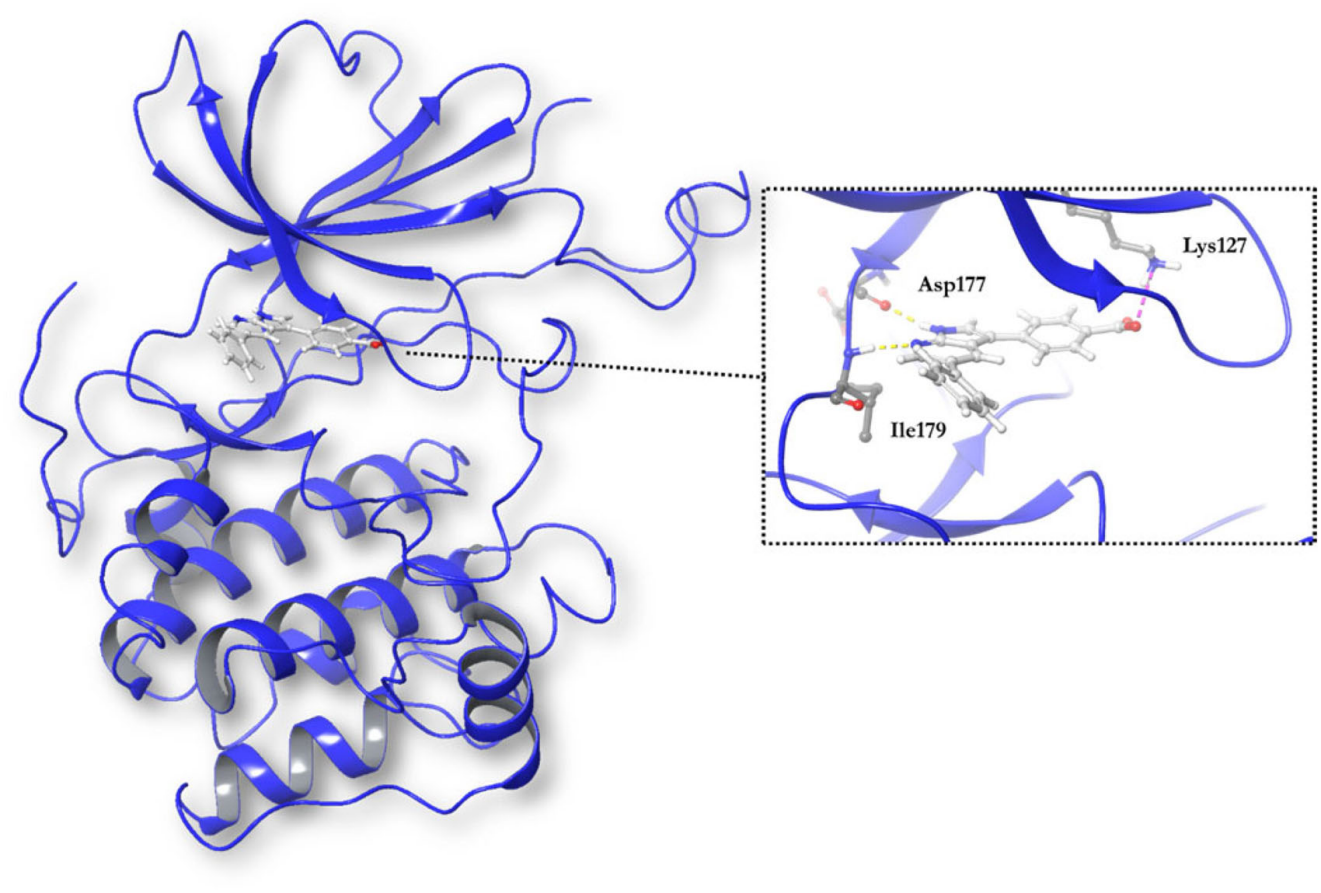
Representation of SGK1 crystalised with the ligand MMG.

Docking studies in the ATP binding site or catalytic site were done using the software Glide (Schrodinger)^31^. The co-crystallised ligand, 4–(5-phenyl-1H-pyrrolo[2,3-b]pyridin-3-yl)benzoic acid (MMG), is a small molecule very similar to GSK650394, and it was included in the chemical set used in the screening as internal reference. As MMG interacts in the crystal structure with Asp177 and Ile179 by two hydrogen bonds (Figure 4), we considered these two interactions in the virtual screening performed. Only those compounds able to interact with SGK1 by those two bonds were selected.

Results were ranked by the docking score function with XP precision (XP Gscore). The MMG reference compound presented a docking score of −11.261 kcal/mol. It is well known that empirical scoring functions in docking screenings are far from precisely predicting binding affinities due to its simplified nature, as neither hydration, entropy nor protein flexibility are considered. However, they are extremely useful to rank a set of compounds where the top of the table is more enriched with potential active ligands, returning a small set of ligands with the highest possible proportion of active compounds^37^.

All the compounds displayed values lower than the reference, thus indicating the compounds from the MBC were less potent than MMG in their binding to SGK1. However, the binding energies were good enough to identify new inhibitors. In order to stablish a cut off value, we selected a batch of compounds with docking scores lower than −8 kcal/mol based on chemical diversity to be experimentally confirmed. A total of nine compounds, with different chemical scaffolds, were selected for the next step (Table 1).

**Table 1.** Compounds selected from the target-based screening based on their docking score (XP Gscore) and structural diversity.

Compound	XP Gscore (kcal/mol)	Structure	Compound	XP Gscore (kcal/mol)	Structure
MMG	−11.3	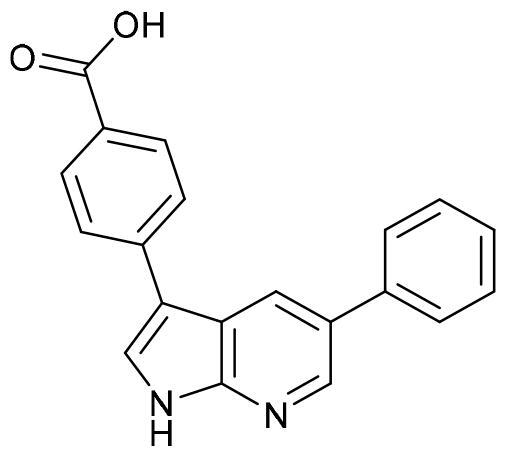	**DM1.55**	−8.4	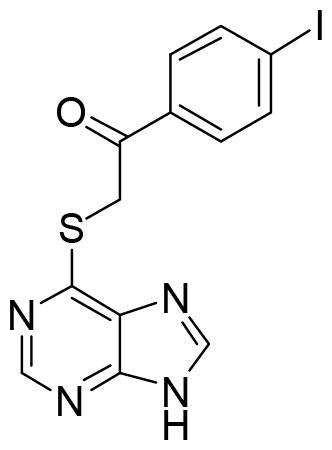
SC695	−8.9	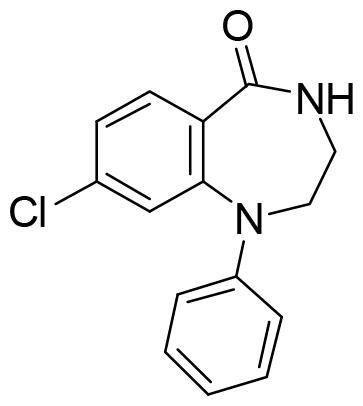	**IGS4.18**	−8.3	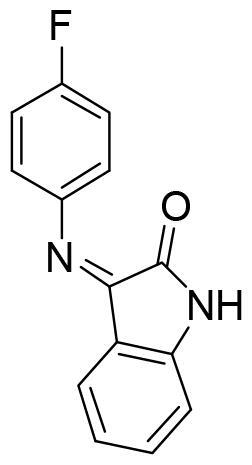
VNG1.6	−8.9	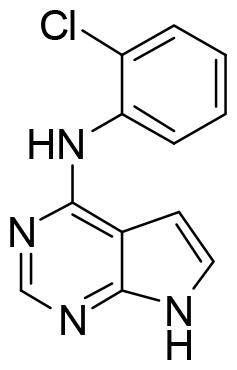	**AM101**	−8.1	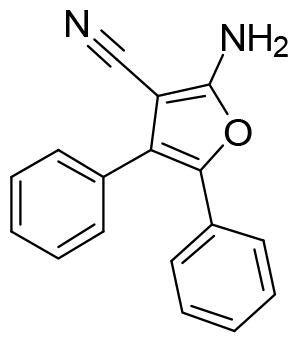
LG1.31	−8.5	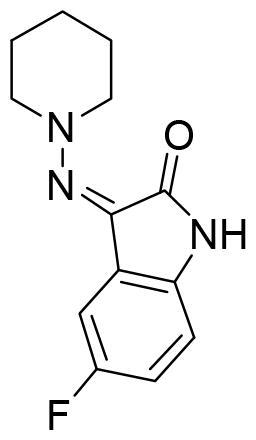	**GMH1.1**	−8.1	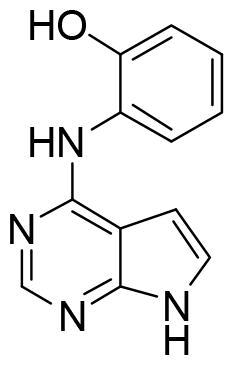
AEL024	−8.5	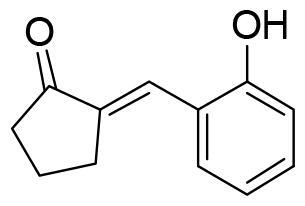	**AC074**	−8.1	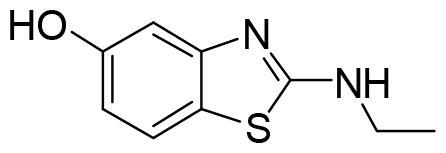

### In vitro evaluation of the compounds

These nine compounds were evaluated against human recombinant SGK1. This biological screening was performed in the MRC Unit of Dundee University using a radioactive methodology with^33^ATP. In addition, the commercial SGK1 inhibitor, GSK650394, was also included in the study as reference standard. Kinase inhibition evaluation was performed in two steps. First, the compounds were assayed at a fixed concentration of 10 µM and only when the kinase inhibition was more than 40% their half maximal inhibitory concentration (IC_50_) was determined. Results are summarised in Table 2.

**Table 2.** Inhibitory values of compounds against SGK1.

Compound	SGK1 % Inhibition@10 µM	IC_50_ (µM)
**GSK650394** ^a^	92.6	0.1
**SC695**	3	–
**VNG1.6**	48	5.2
**AEL024**	1	–
**LG1.31**	5	–
**DM1.55**	7	–
**IGS4.18**	1	–
**AC074**	2	–
**GMH1.1**	28	–
**AM101**	1	–

^a^IC50: 62 nM^15^

**Figure 5.**
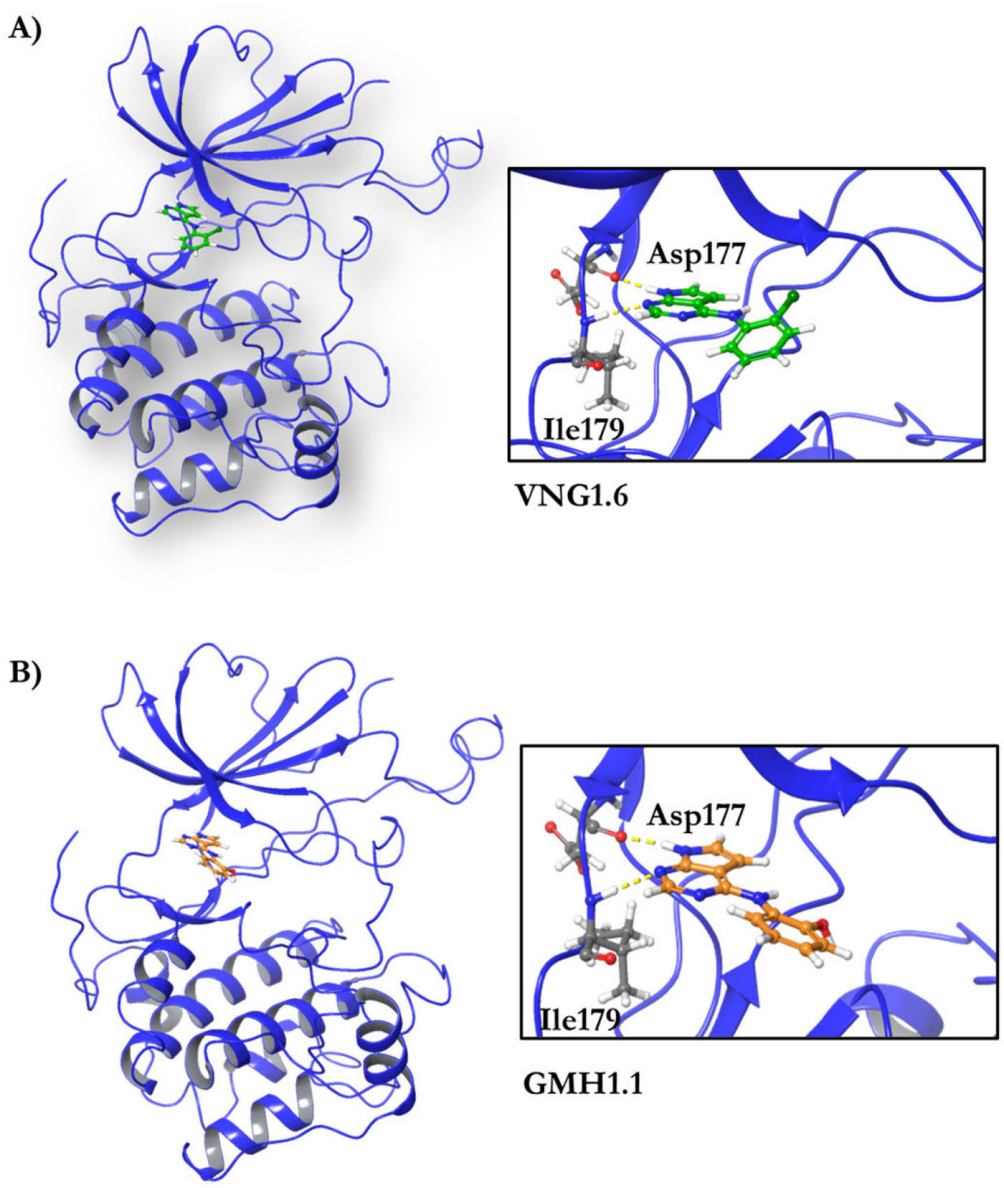
Representation of the binding mode of A) VNG1.6 and B) GMH1.1 in the catalytic site of the crystallised protein SGK1 (3HDM).

Compound VNG1.6 showed an IC_50_ value of low micromolar (5.2 µM), followed by GMH1.1 with a 28% of inhibition at 10 µM. Curiously, these two compounds shared the same heterocyclic scaffold of deazapurine. The binding mode of these two compounds in the catalytic site of the kinase was explored. The deazapurine derivatives, VNG1.6 (Figure 5(A)) and GMH1.1 (Figure 5(B)), bound the kinase like the co-crystalised ligand, MMG, by two hydrogen bonds with Asp177 and Ile179.

Next, we proceeded to select a focussed subset of compounds from MBC library structurally similar to these two compounds. Nine compounds were selected and experimentally evaluated (Table 3). In almost all the cases, we obtained moderate SGK1 inhibitors with IC_50_ values ranged from 3 to 10 µM.

**Table 3.** Inhibitory values of deazapurine related compounds against SGK1 and their IC_50_.

Comp.	SGK1 % Inh@ 10 µM	IC_50_ (µM)	Structure	Comp.	SGK1 % Inh @10 µM	IC_50_ (µM)	Structure
**GSK650394**	92.6	0.1	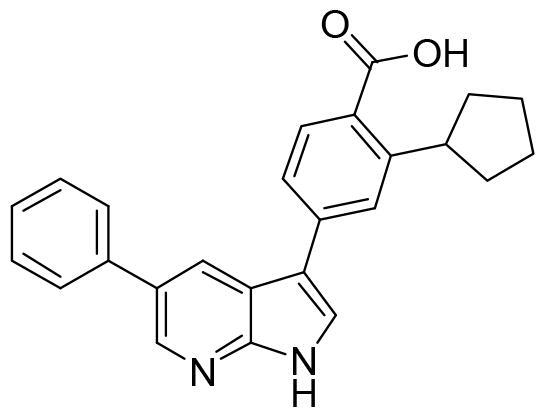	**GMH1.12**	74.3	5.5	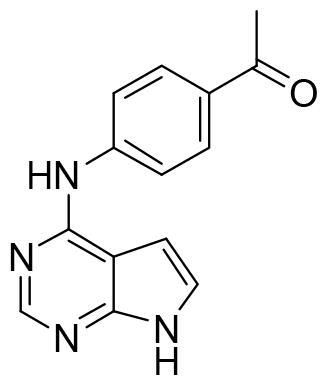
**VNG1.7**	54.0	9.3	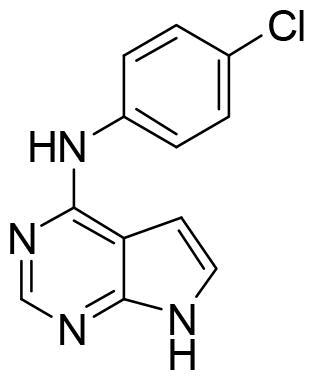	**ERP2.14**	52.6	6.4	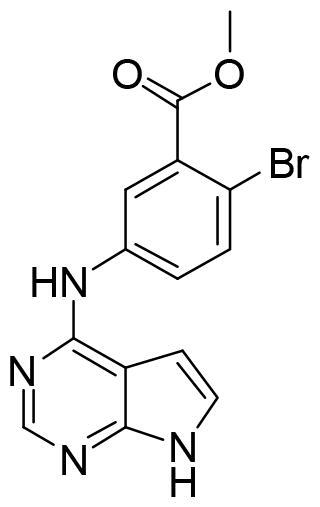
**VNG1.52**	60.5	2.4	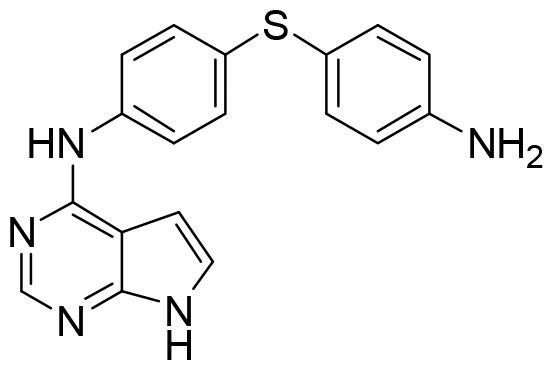	**ERP1.32**	6.6	–	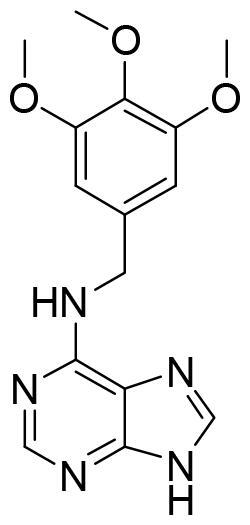
**VNG1.65**	62.6	3.4	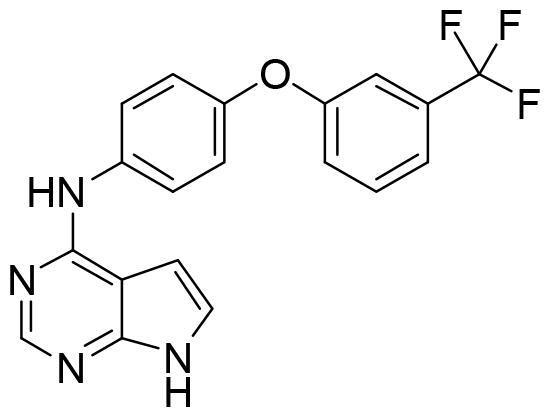	**ERP1.22**	21.9	–	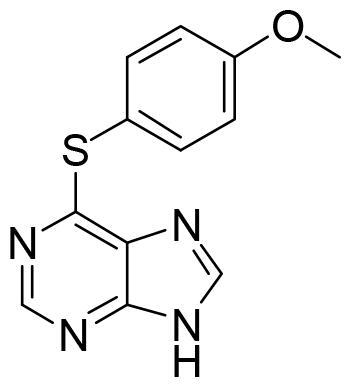
**AEV1.40**	65.3	6.5	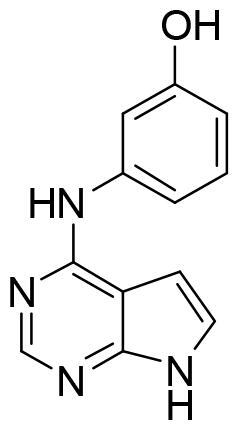	**ERP1.19**	3.9	–	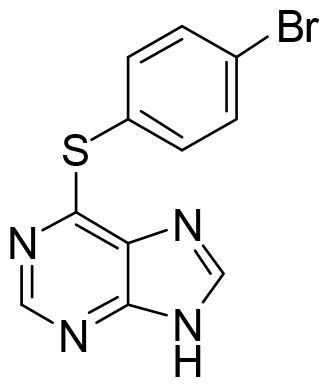

**Figure 6.**
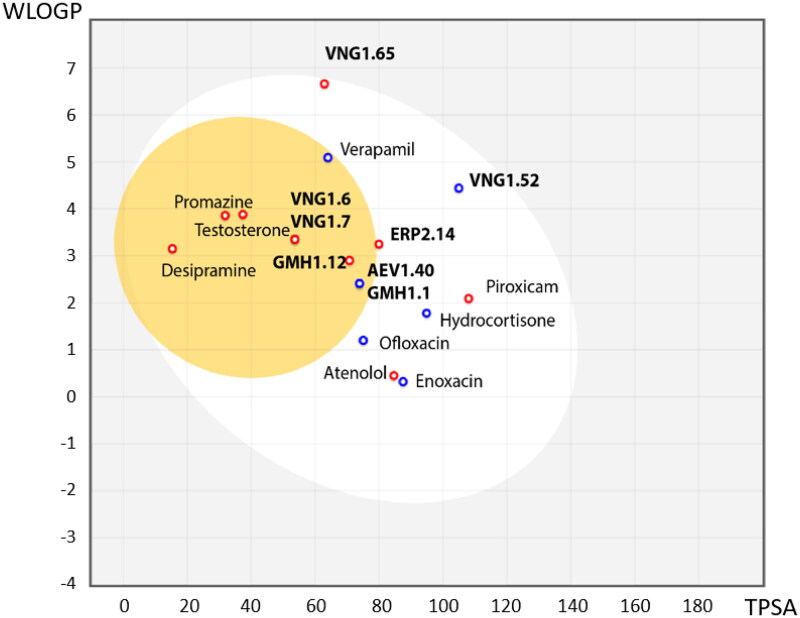
BBB penetration of SGK1 inhibitors from the MBC library according to the BOILED-egg model. WLOGP: Wildman and Crippen log P (n-octanol/water partition coefficient); TPSA: topological polar surface area.

Following the same scheme and using SwissADME tool, BBB penetration was calculated for this family of compounds able to modulate SGK1 (Figure 6). Compounds VNG1.52 and VNG1.65 were located in the gastrointestinal absorption region, ERP2.14 in the limit of both spaces and the rest of the compounds were located in the chemical space predicted to reach the CNS. These results confirm that the deazapurine moiety may be a privileged scaffold for SGK1 inhibition in the brain.

**Figure 7.**
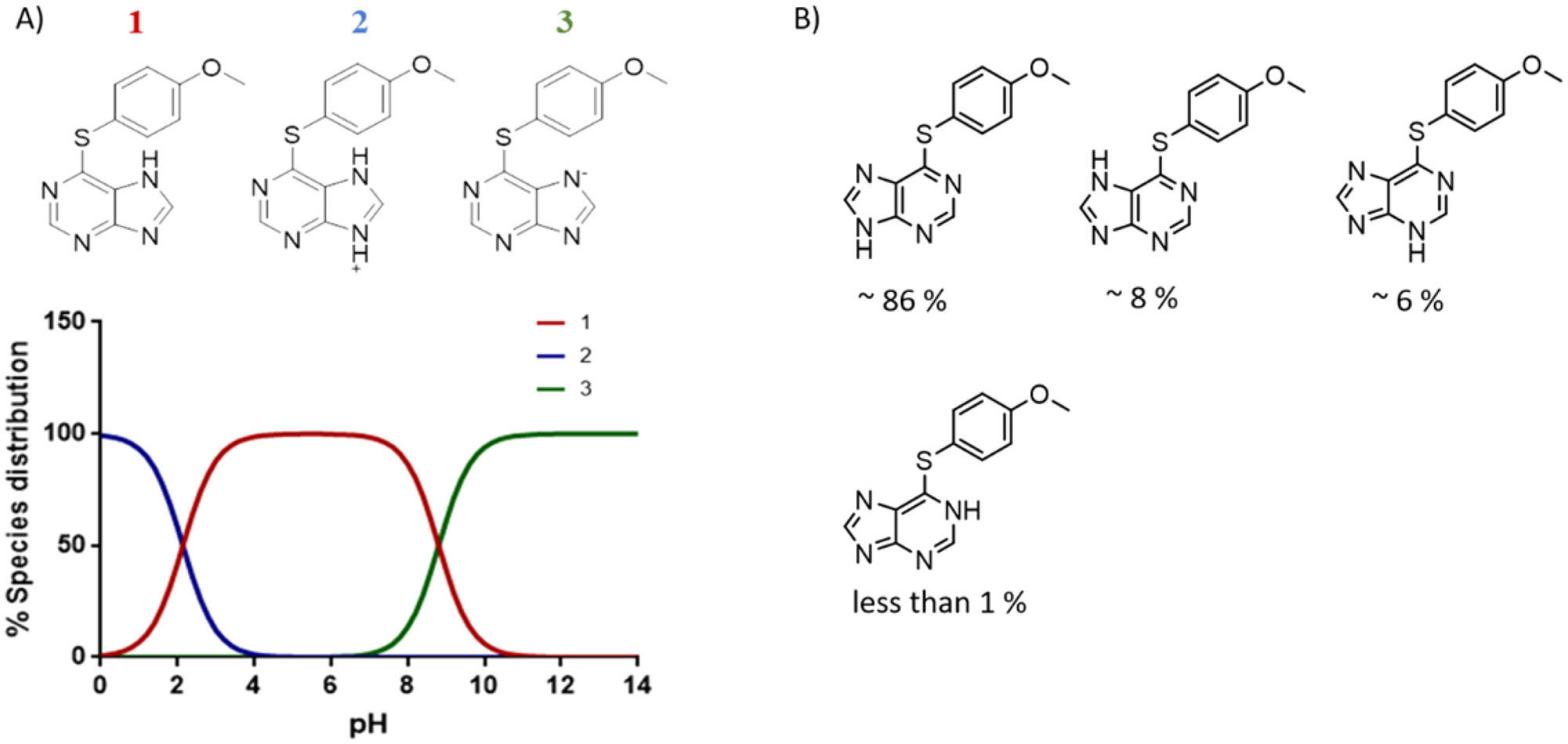
ERP1.22 species distribution. A) pH dependent distribution of the ligand species. B) Tautomeric population of the ligand at its neutral ionisation state.

It was interesting to see the lack of inhibitory activity of the purine derivatives, probably caused by the tautomerism between both nitrogen atoms of the imidazole ring. As we mentioned before, the hydrogen bond interaction between the NH from the heterocyclic scaffold and Arg177 is key for SGK1 inhibition. Thus, in the purine moiety the hydrogen atom can moved between the two nitrogen atoms of the five membered ring. With the software MarvinSketch (version 18.10.0, ChemAxon Ltd.) we could determine that at physiological pH the most abundant species of ERP1.22 is number 1 (Figure 7), the neutral state Using the same software, we calculated the relative abundance of each tautomer for this state. Surprisingly, only the 86% of the population corresponds to the tautomer that is able to interact properly with the hinge region, while the rest of specieslack the hydrogen needed to form the hydrogen bond with Asp177 in the catalytic site. This may decrease the interaction with the two key amino acids within the catalytic site, which translates in the lack of inhibitory potency. Furthermore, in terms of binding energy, desolvation energy plays a critical role in protein-ligand interaction. As the scaffold needs to be almost completely desolvated to reach the hydrophobic binding site, it is very likely that the deazapurine moiety is energetically more favourable to accomplish this in comparison to the purine scaffold. However, further calculations are needed to confirm this.

### Phenotypic evaluation of new hits in mitophagy assay

As it has been previously mentioned, there are controversial results around the role of this kinase in the process of autophagy. Thus, we used an established mitophagy phenotypic assay to evaluate if SGK1 inhibition has a role in the selective degradation of mitochondria by autophagy.

**Figure 8.**
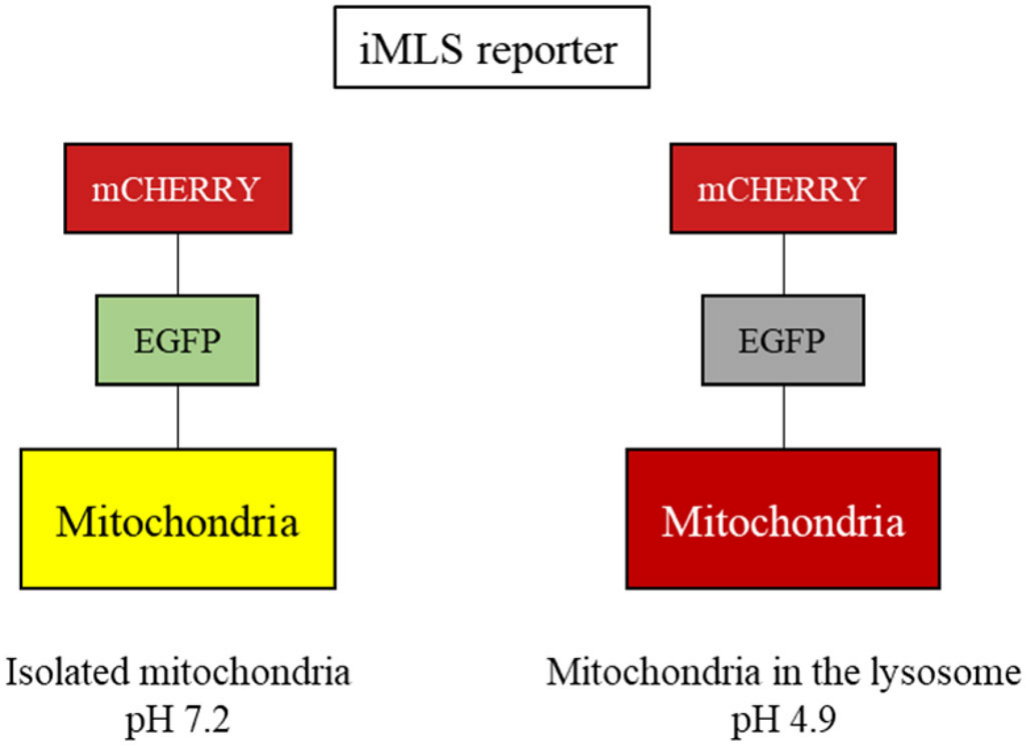
Description of the iMLS reporter.

This phenotypic assay consisted in an image-based assay using the human osteosarcoma cell line, U2OS, with doxycycline-inducible expression of the double-tagged NIPSNAP internal mitochondrial localisation signal (iMLS, NIPSNAP1_1-53_-EGFP-mCherrry) previously described as a mitophagy reporter^33^. We used U2OS cells for receptor-mediated mitophagy, as they do not express PRKN^38^, and U2OS-iMLS cells transduced with a lentiviral particle expressing PRKN (U2OS-iMLS-PRKN) for PRKN-mediated mitophagy. In that way, we were able to assess effects of SGK1 inhibition on both types of mitophagy. Like other tandem reporters, iMLS is pH sensitive (Figure 8). When mitochondria are in the cytosol (pH ∼7.2), the reporter fluorescence becomes yellow as a combination of fluorophores, mCherry and EGFP. However, when the mitochondria are inside the acidic compartments (pH ∼4.5–5), the reporter fluoresces only in red, due to the EGFP quenching at low pH. To induce the expression of the mitophagy reporter, cells were treated with doxycycline for 24 h.

**Figure 9.**
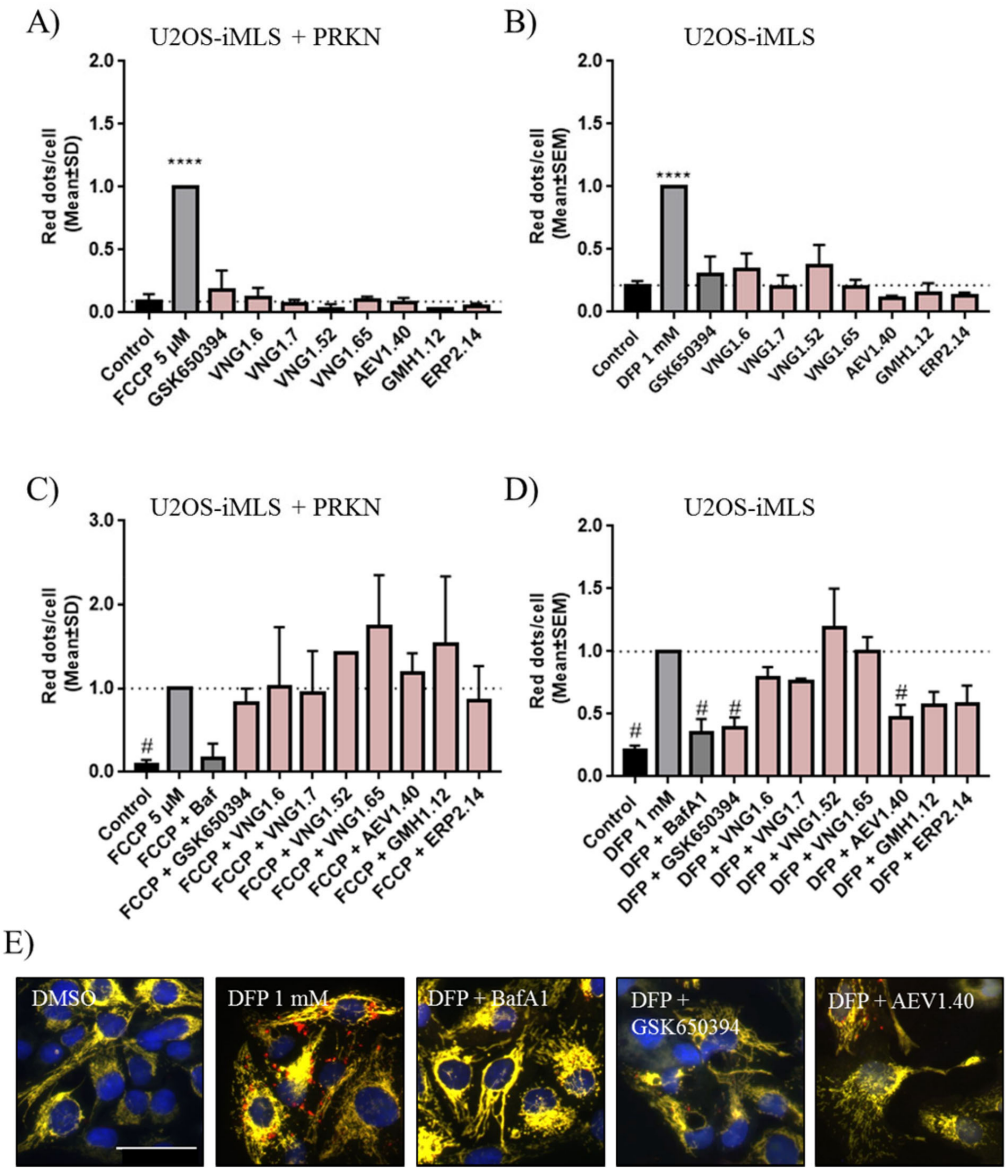
Mitophagy modulation by SGK1 inhibitors. (A,B) Basal and (C,D) induced mitophagy were measured in U2OS-iMLS PRKN and U2OS-iMLS cells treated with the compounds at 10 µM and/or the inducers FCCP and DFP for 24 h. (E) Representation of U2OS-iMLS cells treated as indicated. Scale bar = 50 µm. Data represent the mean ± SD of three replicates in (A–C) and the mean ± SEM of four independent experiments in (B–D). (Significance was determined by one-way ANOVA followed by Dunnett’s multiple comparison test to control, where ****p < 0.0001 versus control in (A,B) and * *p* < 0.05 versus FCCP or DFP in (C,D), respectively).

Thus, both cell lines were treated with the compounds and the internal controls, carbonyl cyanide-p-trifluoromethoxyphenylhydrazonedeferiprone (FCCP) and deferiprone (DFP) to induce mitophagy in a PRKN-dependent and independent manner, respectively (Figure 9(A,B)). Results showed that only FCCP or DFP, induced mitophagy, but neither GSK650394 nor our compounds enhanced the pathway. Then, we co-treated the cells with the mitophagy inducers (FCCP or DFP) and the compounds to evaluate if SGK1 inhibition reduced mitophagy (Figure 9(C,D)). As control standard, we used bafilomycin A1 (BafA1), a V-ATPase inhibitor, which increased the pH, restored EGFP signal in the acidic compartments,^39^ and blocked the fusion between autophagosomes and lysosomes, then inhibiting the final step of the pathway. The mitophagy reduction produced by BafA1 treatment showed that the assay conditions were adequate in both cell lines (Figure 9(C,D)). None of the treatment with SGK1 inhibitors reduced FCCP-induced mitophagy in U2OS-iMLS PRKN cells in Figure 9(C). However, in U2OS-iMLS cells, which lack PRKN, besides BafA1, two compounds reduced mitophagy more than 50%: the commercial inhibitor, GSK650394 and the compound from the MBC library, AEV1.40 (Figure 9(D,E)). Moreover, compounds GMH1.12 and ERP2.14 also decreased mitophagy more than 40% (Figure 9(D)). Interestingly, these three compounds from MBC chemical library reduced also basal mitophagy close to half (Figure 9(B)), indicating the trend of these three compounds to inhibit mitophagy. Thus, we can conclude that SGK1 inhibitors may decrease PRKN-independent mitophagy.

**Figure 10.**
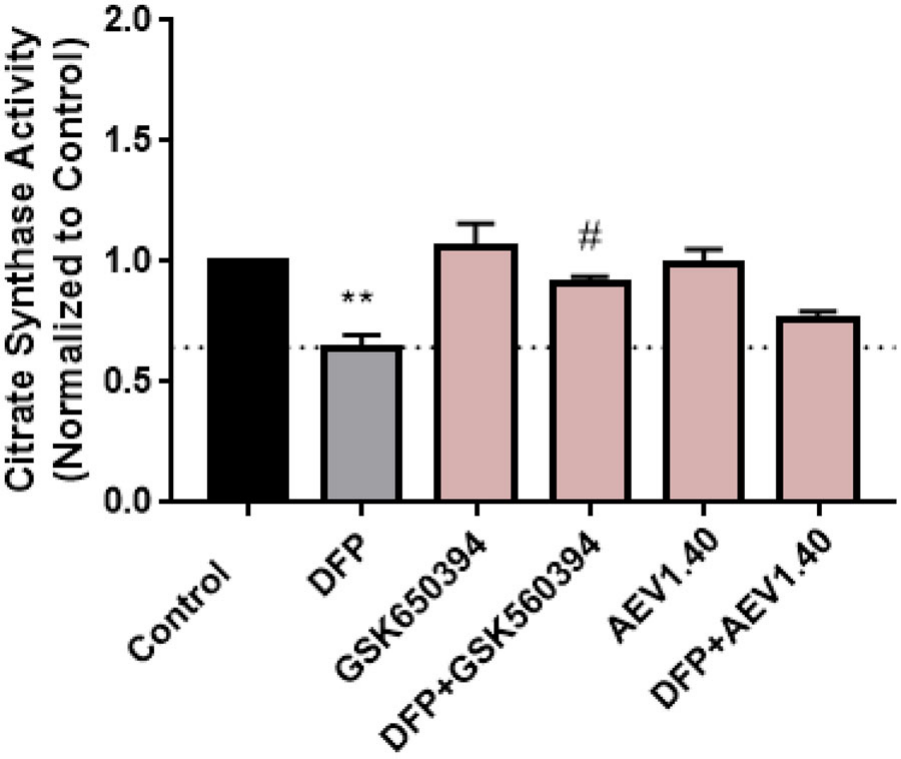
Citrate synthase activity in SH-SY5Y cell line. Cells were treated with DFP 1 mM and/or GSK650394 and AEV1.40 10 μM for 24 h. Data represents mean ± SEM of four independent experiments. (Significance was determined by one-way ANOVA followed by Dunnett’s multiple comparison test to control, where ** *p* < 0.01 versus control and # *p* < 0.05 versus DFP).

We confirmed this effect in the neuroblastoma cellular line SH-SY5Y. For that, the activity of the enzyme citrate synthase was determined. This enzyme participates in the Kreb’s cycle and its activity can be used to measure the amount of mitochondria in a sample. Cells were treated with the inducer DFP and the compounds GSK650394 and AEV1.40. Data showed a decrease in citrate synthase activity when cells were treated with the enhancer DFP, as it reduces the amount of mitochondria by mitophagy. In addition, the co-treatment with the inhibitor of SGK1, GSK650394, significatively recovered the loss of mitochondria to control levels. The compound AEV1.40 showed a similar tendency than GSK650394, increasing the activity of the citrate synthase (Figure 10).

### Therapeutic applicability of SGK1 inhibitors

As SGK1 is reported to be involved in tau phosphorylation^13^, we decided to study our SGK1 inhibitors in a cell model of tau phosphorylation. We used the okadaic acid (OA) model. This toxin is widely used for generating a cellular model to study AD. OA inhibits the activity of serine/threonine phosphatases 1 and 2 A, which leads to an imbalance in phosphorylation homeostasis and, subsequently, increase the phosphorylation of tau protein^40^. This hyperphosphorylation causes cell death. Neuroprotection from this insult was determined with our SGK1 inhibitors treatment.

**Figure 11.**
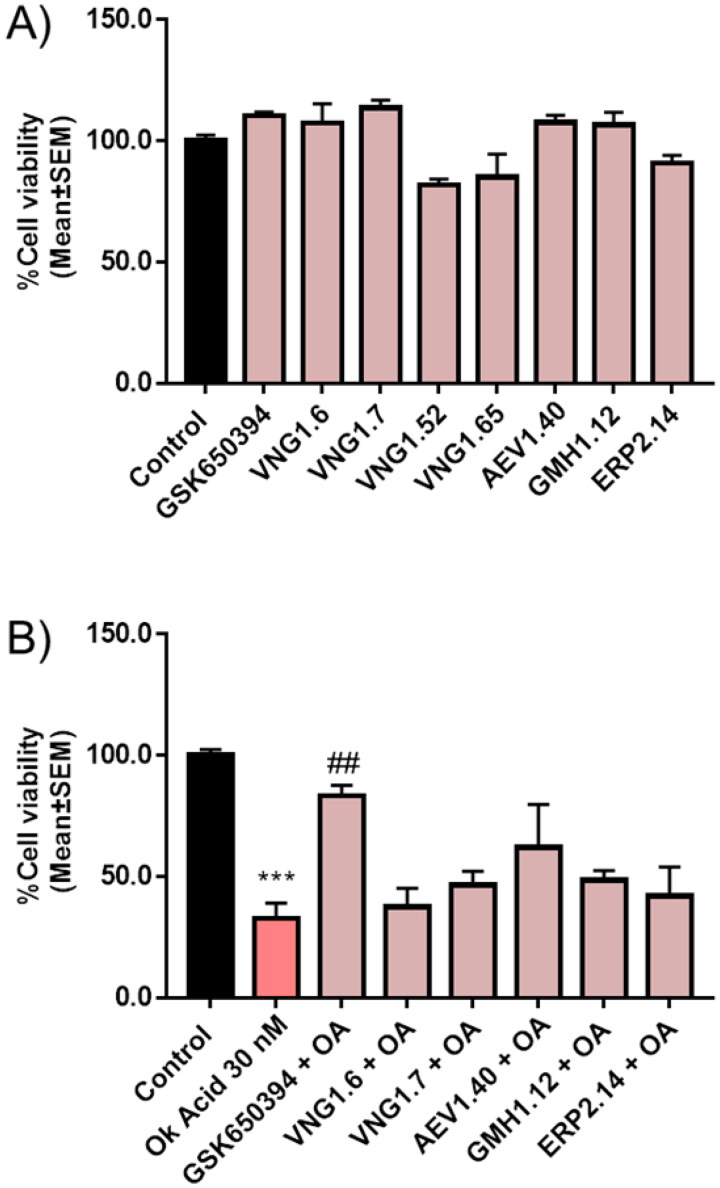
Neuroprotective effect of SGK1 inhibitors in AD cellular model. A) SH-SY5Y cells were treated with the compounds at 10 µM for 24 h. B) Cells were pre-treated with the compounds at 10 µM for 1 h followed by the treatment with OA 30 nM for 24 h. Data were normalised to control and represent mean ± SEM of three independent experiments. (Significance was determined by one-way ANOVA followed by Dunnett’s multiple comparison test to control, where *** *p* < 0.001 versus control and ## *p* < 0.01 versus OA).

First, we measured cell viability in the neuroblastoma cell line SH-SY5Y treated with the different compounds at 10 µM for 24 h (Figure 11(A)). Results showed a slightly decrease in cell viability upon the treatment with VNG1.52 and VNG1.65, the predicted compounds that do not penetrate the BBB. Thus, they were discarded from next steps. Then, SH-SY5Y cells were treated with OA. In fact, a decrease of almost 65% in cell viability was obtained after the treatment with OA 30 nM for 24 h (Figure 11(B)). Thus, we pre-treat cells with the SGK1 inhibitors before OA treatment to evaluate the ability of the compounds to neuroprotect against the toxin. Interestingly, the pre-treatment with GSK650394 protected cells from death caused by the toxin. In addition, the pre-treatment with AEV1.40, GMH1.12 and ERP2.14, which also inhibited or tended to inhibit mitophagy, reduced the cell death caused by OA.

**Figure 12.**
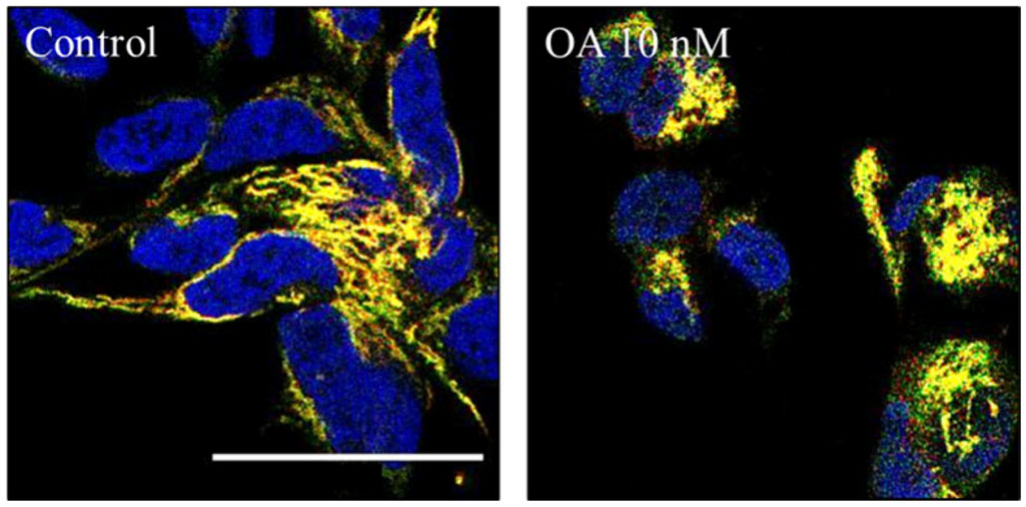
Mitophagy modulation by OA. A) Representation and quantification of SH-SY5Y MitoQC cells treated with OA 10 nM. Data represent mean ± SD of replicates from one experiment. Scale bar = 30 µm.

Finally, we wondered if OA was modulating mitophagy. To test that we used SH-SY5Y cells expressing another mitophagy reporter bound to the outer mitochondrial membrane protein FIS1 (MitoQC)^34^. SH-SY5Y MitoQC cells were treated with OA to evaluate the influence of OA on mitophagy. The concentration of the toxin was reduced from 30 nM to 10 nM due to the high cell death produced by the highest concentration in this cell type. However, SH-SY5Y MitoQC cells did not show any mitophagy modulation when they were treated with OA (Figure 12). Thus, we could confirm that both, OA cell death and its rescue with GSK650394 must be independent of the mitophagy pathway.

All together, these results show that SGK1 inhibitors decreased mitophagy in cells without PRKN. However, the tendency of these compounds to rescue from OA-cell death was independent of their role in mitophagy in SH-SY5Y cells. Thus, as OA is known to increase the phosphorylation of tau, the rescue of cell death produced by the SGK1 inhibitors may be related to a decreased in tau phosphorylation. As mentioned previously SGK1 phosphorylates tau, and its inhibition could also contribute to decrease that pathological hallmark of AD.

## Conclusions

The new role of the kinase SGK1 in neurodegeneration and the lack of BBB-penetrant inhibitors made us to initiate a drug discovery program to find new SGK1 modulators that reach the CNS. By means of a virtual screening approach and a subsequent compound selection based on their chemical structure, we have identified some molecules belonging to the deazapurine family as inhibitors for this kinase. Furthermore, these compounds are predicted to cross the BBB, being an advantageous starting point for developing new drugs that reach the CNS. Moreover, SGK1 inhibition by these molecules showed neuroprotection against the OA model of AD and modulate the mitophagy activity U2OS-iMLS cells. Thus, we propose the deazapurine moiety as a potential starting point to continue working on SGK1 trying to decipher its role in neurodegeneration.
